# Palliative Dialysis: A Change of Perspective

**DOI:** 10.14740/jocmr1773w

**Published:** 2014-05-22

**Authors:** Thiago Gomes Romano, Henrique Palomba

**Affiliations:** a Nephrology Department, ABC Medical School, Brazil; b Sirio Libanes Hospital, Intensive Care Unit, Brazil; c Sancta Maggiori Hospital, Brazil; d Albert Einstein Hospital, Intensive Care Unit, Brazil

**Keywords:** Palliative, Dialysis, Elderly

## Abstract

The aging phenomenon of dialysis patients is a worldwide reality, observed in developed and developing countries. Those patients have high incidence of chronic conditions along with high mortality rates and for some of them a decline in functional status within the first 12 months of dialysis therapy. Nevertheless, the elderly dialysis patients represent a very heterogeneous group where prognostic tools may help the decision-making process together with family members, medical staff and the patients. Despite the fact that there are many validated prognostic tools in elderly population, no score has the aim to guide the decision to withhold or withdrawn the dialysis procedure; therefore, in many cases, a time-limited trial is supported. After the failure of improvement in life quality and certitude of the poor prognosis, the withdrawing from renal replacement therapy can be done. Medical literature, from developed countries, brings robust evidence that the process of withdrawing the dialysis procedure, after a fail in the so-called “time-limited trial”, along with good quality palliative care in this scenario is related to a good quality of death. We, on the other hand, believe that the withdrawing process in countries where hospice and good palliative care is not a reality may be associated with bad outcomes. Therefore, this review discusses a way to improve end-of-life symptoms in countries where palliative care facilities are not a reality, the so-called “palliative dialysis”.

## Introduction

In Western countries, the demand for dialysis is increasing in elderly patients [1, 2]. The United States Renal Data System indicated an increased diagnosis of end-stage renal disease among persons aged > 65 years and especially among those aged > 80 years [3]. Data from the European Renal Association-European Dialysis and Transplant Association demonstrated that the incidence of patients aged > 65 years increased from 22% in 1980 to 55% in 2005.

The aging phenomenon of dialysis patients is also observed in developing countries. According to the annual census from the Brazilian Nephrology Society, approximately 36% of dialysis patients are older than 65 years.

Along with this aging population comes the prevalence of chronic conditions, such as diabetes, hypertension and cardiovascular diseases [4], which result in high mortality rates of approximately 23% per year [5].

The burden of symptoms and low quality of life are noted in this scenario [6]. A high proportion of patients regret their decision to start dialysis [7]. Somatic and psychosocial conditions are frequent, and the prevalence of most geriatric conditions is comparable to those observed in elderly cancer patients [8].

According to similar well-based evidence in the medical literature, the functional status in subgroups of patients, such as nursing home residents, declines during the first 12 months after the initiation of dialysis therapy [9].

Another important topic is the role of the family and caregivers in the process of renal replacement therapy (RRT). Parlevliet et al have shown that 84% of caregivers felt overburdened by the situation of their family members [8]. Frequently, the caregivers were obliged to change their routine and professional schedules to assist their loved ones.

Despite this knowledge and the fact that most of the patients demand access to their prognostic information, nephrologists do not frequently release this type of information [7, 10].

The elderly dialysis patients comprise a heterogeneous group; however, young patients can have worse functional status than most elderly people. Therefore, the decision-making process with regard to RRT, which involves family members, the medical staff and the patients, demands knowledge of the some prognostic tools.

## Prognostic Tools

Most of the prognostic tools take into account the presence of comorbidities and geriatric conditions, such as dementia and frailty.

Chandna et al, in a cohort study of 844 patients, demonstrated that the benefits of dialysis therapy, within the limits of mortality, in individuals above 75 years old with multiple chronic conditions seem to be small [11]. In Europe, a proposal called “Maximum Conservative Management” (MCM), which is based on a multidisciplinary approach with nutritionists, social workers, psychologists and other health professionals, is defended in certain cases [12].

Reports have documented that patients who receive dialysis have higher survival rates than those who receive MCM; however, this survival benefit results in more hospitalization and a higher rate of in-hospital death (65%) than those who receive MCM (27%) [13].

Another prognostic tool is frailty. Frailty is defined as a multidimensional construct that reflects the decline in health and functioning observed in the elderly, ultimately resulting in increased risk for disability, hospitalization, institutionalization and death [14]. We can screen for frailty using simple criteria, such as the presence of unintentional weight loss, slow walking speed, weakness, exhaustion and low level of physical activity [15]. Frailty is reportedly a good prognostic tool in patients who receive dialysis. The prevalence of frailty in dialysis patients is approximately 67% and bears twice the chance for death and hospitalization [16].

Couchoud et al, in a study using the data from de French Rein registry from 2002 to 2006, developed a prognostic score that was validated for individuals older than 75 years. Factors associated with higher mortality rates during the first 6 months of dialysis therapy include the following: the presence of diabetes, congestive heart failure (NYHA III or IV), peripheral vascular disease (stage III or IV), arrhythmia, active malignancy, body mass index of over 18.5 kg/m^2^, severe behavioral disorder, dependency on transfers and initiation of unplanned dialysis [17].

Similarly, the Palliative Care Department of King’s College demonstrated that comorbidity and ischemic heart disease are associated with reduced benefits in terms of mortality of the RRT in individuals older than 75 years [18].

Analyzing the retrospective data of 272,000 patients of the US Renal Data System, Rakowski et al studied dementia as a prognostic tool. They observed that the diagnosis of dementia was associated with a 24% survival rate compared with a 66% survival rate of individuals who had no cognitive impairment (P < 0.001). The median time to death in the first group with dementia was 1.09 years versus 2.7 years in the second group with no cognitive dysfunction (P < 0.001) [19].

The nurses’ impression has been studied in this scenario. In a published study, Moss et al queried professionals involved in dialysis therapy and management and asked “Would I be surprised if this patient died in the next year?” The negative answer, along with measurements for serum albumin, age, peripheral vascular disease and dementia, was a reliable and quick screening tool to determine the patient’s risk for mortality [20].

The medical literature has robust evidence regarding the prognostic factors in elderly dialysis patients. However, the role of a prognostic score is not to guide therapy but to serve as a critical tool, among other factors, to assist in the decision-making process.

## Decision-Making Process

In 2000, the Renal Physicians Association and the American Society of Nephrology, in conjunction with representatives from multiple disciplines and organizations in the dialysis community, kidney patients and family members, internal medicine physicians as well as a bioethicist and a public policy expert comprised a working group that formulated “Clinical Practice Guideline on Shared Decision-Making in the Appropriate Initiation of and Withdrawal from Dialysis” [21]. This guideline includes nine recommendations regarding issues on prognostic information, informed consent or refusal, palliative care and conflict resolution. This document proposes that “for patients requiring dialysis, but who have an uncertain prognosis or for whom a consensus cannot be reached about providing dialysis, nephrologists should consider offering a time-limited trial”. After the quality of life fails to improve and there is certainty of poor prognosis, patients should be withdrawn from dialysis.

This approach seems reasonable. Cohen et al conducted a prospective cohort study involving six dialysis clinics in the US and two clinics in Canada; 131 adult patients who received maintenance dialysis died after treatment was withheld. They reported that 38% of the family members and caregivers considered that their relatives had a “very good death”, 47% related a “good death” and only 15% felt it was a traumatic process [22].

The acceptance of this practice by the nephrology community is also changing. In France, a physician made the decision to stop dialysis approximately 77% of the time [23]. Using an online survey, Holley et al published that in 1990, approximately 39% of nephrologists would stop dialysis in a severely demented patient compared to 53% in 2005 (P < 0.00001); the same doctors were more likely to honor a dialysis patient’s do-not-resuscitate order (30% in 2005 versus 15% in 1990) (P < 0.0002) [24].

One essential explanation for the data described may be the maintenance of good quality care that is provided to patients who have dialysis therapy withdrawn. Approximately 42% of the patients who had their dialysis suspended in the US had access to hospice facilities. This access was shown to be good for patients, their families and the public health system with reduced costs. The median cost per patient for end-of-life care in hospice facilities was US$ 1,858 versus US$ 4,878 for patients cared for in facilities outside hospice (P < 0.001) [25].

Despite its valid concept, approximately 12% of patients are unsure or believe that discontinuing dialysis is the equivalent to suicide [26], at the same time patients seem to trust the dialysis team regarding the end-of-life decision, 55% depend on the social and emotional support provided by their physician during their illness, 65% are comfortable discussing end-of-life issues with the nephrology staff and 83% feel it is extremely important to be prepared in case of death, according to a survey design by Davison [7]. People are not always consistent about their wishes, and they may change their minds depending on their current health state [27]. Principles of a good death include having the time to say goodbye, controlling pain and other adverse symptoms and receiving spiritual and emotional support. In several cases, especially in countries where hospice care is not a reality, we believe that a gray-scale option seems more reasonable.

## Reality of Withdrawing Dialysis in Developing Countries

Most data addressing the quality of death after the withdrawal of dialysis come from countries where hospice and palliative care are well established. After a 2-year analysis of the US Renal Data System, Murray et al demonstrated that 21% of 115,239 patients died after dialysis suspension and that 41.9% of those patients used hospice facilities with cost reduction from the public health system [25].

Many countries do not share the same reality. The Worldwide Palliative Care Alliance in 2006 published a document that divides countries into four-part typology that depicts levels of hospice and palliative care development. Most countries are categorized globally as having localized provision, many are categorized as having capacity building and few of them are categorized as having no activity yet identified. Data from Brazil show an indicative ratio of palliative care services to a population of 13,315,000 people per palliative care service.

We therefore believe that in this scenario, the withdrawal from dialysis may not be the best choice.

## Concept of Palliative Dialysis

We believe a conduct that allies the mechanical support aimed at symptom relief over classical therapeutic goals with the improvement of palliative care through continuous educational programs for the nephrology staff is a first step for changing the facts described above.

In 1998, Eibach and Schaefer described the concept of basic needs and ethical issues in dialysis [28]. Those so-called basic needs are those that a newborn is unable to provide for himself, such as nourishment, cleanliness, bedding, relief from pain and human care. In patient care, the provision of fluid and nutrition is not given with the intention of treating the disease, but fluid and nutrition are given to satisfy basic needs, which cannot be withheld from any human. In the article by Eibach and Schaefer, the maintenance of mechanical support, basically through ultrafiltration, is the mainstream for providing human basic needs in end of life.

Our concept of palliative dialysis proposes a change of perspective in treatment goals. This change may be achieved by ultrafiltration alone or by dialysis itself, depending on the clinical perspective.

Designed in 12 countries to produce treatment targets in chronic dialysis patients, the Dialysis Outcomes and Practice Patterns Study showed that in most countries, there was no difference in the prescribed treatment time normalized by body weight for the elderly versus individuals aged < 45 years [29]. These data alert us that targets, such as dry weight, Kt/V and serum phosphorus, are still aimed in elderly patients, which may not be the primary objective for several of them.

The palliative dialysis concept proposed in this paper is in accordance with the World Health Organization definition of palliative care, which is “An approach that improves the quality of life of patients and their families facing the problems associated with life-threatening illness, through the prevention and relief of suffering by means of early identification and impeccable assessment and treatment of pain and other problems, physical, psychosocial and spiritual”.

One simple assessment instrument tool for quality palliative care is the PEACE tool that addresses six domains: physical symptoms, emotive, autonomy-related issues, communication and completion of life affairs-related issues, economic burden and other practical issues and transcendent spiritual issues [30]. Individualizing the RRT prescription can improve the physical, emotive and autonomy-related issues.

In the medical literature, there are several reports on the incidence of symptoms in advanced renal disease that is managed without dialysis. Murtagh et al reported that lack of energy, drowsiness, dry mouth, dyspnea, pain, sleep disturbance, restless legs, pruritus, dry skin and constipation are frequently observed symptoms of severe renal disease [31]. Murphy et al described the same symptoms in another cohort of patients with chronic kidney disease who were managed conservatively [32]. The most relevant symptom that palliative dialysis can aid is dyspnea, which is caused by either fluid overload or acidosis. Other symptoms can be treated with medications. The impact of dialysis in the emotive domain must be extremely individualized. Some patients believe that the continuation of dialysis is a way to receive medical care and that the dialysis room provides an occasion for social interaction. The extremely individualized terminology used above is based on the data from the paper of Morton et al; some patients were willing to forgo 7 months of life expectancy to reduce the number of required visits to the hospital, whereas other patients were willing to forgo 15 months of life expectancy to increase their ability to travel [33].

Beyond the domains of the PEACE tool, the most important factor is the autonomy of the patients who face severe chronic kidney disease and the relatives who provide support.

We realize that the delivery of palliative care in this scenario should not start during the last moments of the patients’ lives; palliative care must be a part of the treatment from the beginning of dialysis therapy. This concept is true for any chronic disease, such as chronic obstructive pulmonary disease or heart failure [34]. To implement palliative care in this setting, the clinical staff in the dialysis unit must be trained and familiar with this concept. Additionally, they must incorporate palliative care into the nephrology residency training and teaching programs.
